# Internet use and cognitive frailty in older adults: a large-scale multidimensional approach

**DOI:** 10.1007/s10433-022-00686-2

**Published:** 2022-03-02

**Authors:** Samantha Dequanter, Ellen Gorus, Sven Van Laere, Nico De Witte, Dominique Verté, Iris Steenhout, Maaike Fobelets, Ronald Buyl

**Affiliations:** 1 Department of Public Health Sciences, Biostatistics and Medical Informatics (BISI) Research Group, Vrije Universiteit Brussel, Brussels, Belgium; 2 Department of Gerontology, Frailty in Ageing (FRIA) Research Group, Vrije Universiteit Brussel, Brussels, Belgium; 3 Department of Geriatrics, UZ Brussel, Laarbeeklaan 101, 1090 Brussels, Belgium; 4 Department of Educational Sciences, Belgian Ageing Studies (BAS) Research Group, Vrije Universiteit Brussel, Brussels, Belgium; 5 Department of Education, Health and Social Work, University College Ghent, Ghent, Belgium

**Keywords:** Aged adults, ICT, Internet, Technology, Cognitive impairment, Frailty

## Abstract

Although research on sociodemographic correlates of internet use in older adults without and with pronounced cognitive impairment is already quite extensive, much less is known about the relationship between cognitive frailty (CF) and this behaviour. As CF is associated to multidimensional frailty aspects, this study explored the relationship between internet use and CF, operationalised as Subjective Cognitive Impairment, in older adults by means of a comprehensive explanatory model including sociodemographic factors and multiple frailty measures. The dataset included a sample of community-dwelling 60 + older adults that were included in the Belgian Ageing Studies (BAS) and that completed survey questions on (i) internet use frequency and (ii) internet activities. Multidimensional frailty was measured with the CFAI-Plus. The analysis comprised a structural equation modelling (SEM) procedure. Internet use was frequent; however, it became less frequent with higher CF. Moreover, the latter used less tablets as compared to the no-low CF group. Navigating the web, sharing email and online banking were the most frequently reported activities. Tele-communicating with Skype, online shopping and using e-government services were the least frequent. Age, female gender, lower income and living with a partner were also negatively associated with internet use. To conclude, CF, along with other frailty and sociodemographic factors, was negatively related to internet use in older adults. Future research should focus, amongst others, on the dynamic processes underlying internet use in the population of older adults affected by CF.

**Supplementary Information:**

The online version contains supplementary material available at 10.1007/s10433-022-00686-2.

## Introduction

As worldwide population ageing has become a well-known phenomenon (World Health Organization [WHO] 2018), the double greying effect resulting from the growing segment of oldest-old (85 and above) is also increasingly observed (Eurostat 2020). Globally, these age-related demographic transitions are causing several societal challenges, including the increasing prevalence of neurocognitive disorders such as Mild Cognitive Impairment or dementia (Prince et al. 2013). Since these cognitive disorders often result in disability and dependence (Sousa et al. 2009) and can cause safety issues (Thoma-Lürken et al. 2018), older adults affected by them are particularly vulnerable for institutionalization (Eaker et al. 2002). As this is an undesirable outcome for most of these older adults (von Kutzleben et al, 2012; WHO 2012), strategies that facilitate ageing at home are needed.

In this light, many approaches have already been developed, including optimization of community care (Dequanter et al. 2020a), innovation of community policies (Alzheimer’s Disease International 2016) and provision of supportive interventions for informal caregivers (Schulz and Martire 2004). In addition, advancements in information and communication technology (ICT) have led to the emergence of complementary support sources. In fact, technological innovations supporting the daily lives of people with dementia are considered integral components in the WHO’s global action plan on the public health response to dementia (WHO 2017). A recent review of technological solutions supporting cognitively impaired older adults revealed a growing research interest in this field and promising effects on a wide range of outcomes (Dequanter et al. 2020b).

Yet, these technological solutions can only be of value if they are used by the targeted population. Studies on internet use among older adults report varying use rates between 13 and 74% (Arcury et al. 2018; Berner et al. 2013; Chang et al. 2015; Choi and DiNotto 2013; Huxhold et al. 2020). Moreover, internet access depends on time and location. A recent 12-year follow-up study by Huxhold et al. (2020) reported internet access to be more than doubled among the group of older adults. With regard to location, older adults living in Northern and Western European countries are more likely to have internet access than those living in other European regions (Huxhold et al. 2020; Vulpe and Crăciun 2020). Other main characteristics associated with internet use in this population are age, gender, education, income, living situation and functional ability (Arcury et al. 2018; Choi and DiNotto 2013; König et al. 2018; Peacock and Künemund, 2007; Quadrello et al. 2005). The most frequently used devices by older adults are mobile phones (91–92%), followed by computers (61–86%) and tablets (39%) (Hernández-Encuentra et al. 2009; LaMonica et al. 2017). A recent study by Stockwell et al. (2020) revealed a relationship between use frequency and ICT device, with the higher frequent users more often using smartphones and tablets as compared to less frequent users, who tend to use computers more often. Sharing email, communicating with family members or friends, reading newspapers and online banking are the most frequent internet activities undertaken by older adults (Chang et al. 2015; Choi and DiNotto 2013; Hernández-Encuentra et al. 2009; König et al. 2018).

Although internet use among the older population is gaining attention, still little is known about the relationship between cognitive impairment and cognitive frailty (CF) on the one hand, and this behaviour, on the other hand. CF refers to a heterogeneous clinical syndrome of cognitive impairment that is linked to physical factors in individuals without concurrent dementia (Kelaiditi et al. 2013; Panza et al. 2015; Ruan et al. 2015). Besides syndromes of objective cognitive impairment, such as mild cognitive impairment (MCI), the clinical concept of CF also refers to subjective cognitive impairment (SCI) (Ruan et al., 2015). While previous studies have already pointed out potential negative effects of lower cognitive ability, MCI and dementia on internet access, adoption and use amongst older adults (Berner et al. 2013; Choi and DiNotto 2013; Elliot et al. 2013; Huxhold et al. 2020; Kamin and Lang 2020), few studies have reported on the effects of SCI on internet use. In a study by LaMonica et al. (2017), groups of older adults with SCI, MCI or dementia were identified and compared on ICT and internet engagement. The results revealed a negative relationship between the degree of cognitive impairment and the use of ICT and internet, with lower computer and smartphone use among more severely cognitively impaired older adults. Nevertheless, recent research findings by Malinowsky et al. (2017) point out that technology use can already be affected in older adults with SCI, as compared to older adults without SCI.

Granted that a reasonable amount of research was already conducted to examine correlates of ICT and internet use in cognitively healthy older adults, as well as older adults with pronounced cognitive impairment (MCI, dementia), the relationship between these behaviours and CF or SCI remains relatively understudied. Nevertheless, as CF can be a precursor of neurodegenerative processes (Kelaiditi et al., 2013), which are well known to negatively impact internet use, research should devote more attention to it. Moreover, as literature considers frailty more and more to be a multidimensional concept, the concept of CF should also be approached in a holistic way, including associations with physical, psychological, social and environmental aspects of ageing (Azzopardi et al., 2016; De Witte et al., 2013; Gobbens et al., 2010; Kwan et al., 2019). Most of these multidimensional frailty domains have been associated with lower internet use among older adults (Choi and DiNotto, 2013; Huxhold et al., 2020; Keränen et al., 2017; König et al., 2018); however, research on these specific relations remains scarce. Therefore, the present study aims to explore how divergent sociodemographic and biopsychosocial characteristics, with a focus on CF, are connected to internet use in older adults.

## Methods

### Design and participants

The present study used data originating from the Belgian Ageing Studies (BAS). The BAS is a continuous large-scale cross-sectional survey study on social policy, feelings of safety and social, cultural and political participation among older adults. Although this paper-based questionnaire is highly structured, it is dynamic in that way that items are added in order to meet the changing characteristics and needs of the older adult population. In each participating municipality, a stratified (gender, age) random sample was drawn from census data of community-dwelling older adults aged 60 and over living in Belgium (BAS, 2013; De Donder et al., 2014). Data are collected since 2004 by means of peer research. Thereby, older volunteers are actively involved in the recruitment of peers and in the data collection process.

For the purpose of this study, a subset of participants who completed all questions on internet use (frequency and type of activities) was selected (*n* = 3019). Included data were collected between 2018 and 2020.

All participants consented to participate in the study before completing the questionnaire. The study was approved by the ethical committee of the Vrije Universiteit Brussel (B.U.N. 143201111521).

### Measures

Sociodemographic characteristics of the participants including age, gender, highest obtained education level (none or primary education, lower secondary education, higher secondary education, higher education), living situation (living with partner/children/grandchildren/parents or others) and net income (less than €1250/month, €1250–2000/month, more than €2000/month) were collected.

To examine biopsychosocial aspects of internet use in the target group, measures originating from the Comprehensive Frailty Assessment Instrument (CFAI-Plus) were used (De Roeck et al. 2018; De Witte et al. 2013, 2018). The CFAI-Plus is a well-developed multidimensional frailty instrument that includes cognitive (CFAI-COG), physical (CFAI-PHYS), psychological (CFAI-PSY), social (CFAI-SOC) and environmental (CFAI-ENV) frailty domains. A detailed overview of the included CFAI-Plus items can be found in Supplementary file 1. CFAI-COG is measured through four items related to subjective cognitive complaints. CFAI-PHYS is operationalized through four items related to limitations in activities of daily living. CFAI-PSY and CFAI-SOC are both measured as combinations of items, with the former comprising five items on mood disorders and three items on emotional loneliness, and the latter comprising three items on social loneliness and one on social support network. Finally, five items on housing and neighbourhood-related aspects refer to the CFAI-ENV domain. All domain scores were recalculated to obtain scores ranging between 0 and 25, with higher scores referring to higher risk of frailty. Obtained CFAI-COG domain scores correspond to three levels of CF: no-low (0.00–3.13), mild (4.69–9.38) and high (10.94–25.00) CF, as proposed by De Roeck et al. (2018).

Internet use was operationalized as a combination of items referring to ICT and internet use. The frequency of internet use (IU frequency) was measured on a 5-point Likert scale, ranging from ‘never’ to ‘multiple times a day’. The type of internet activity (IU activity) was measured by means of nine dichotomous variables (yes/no) referring to (i) navigating/searching the internet, (ii) sharing email, (iii) using e-government services, (iv) maintaining contact with (grand)children, (v) social media, (vi) tele-communicating with Skype, (vii) online shopping, (viii) online banking and (ix) online administration (e.g. registering subscriptions, buying tickets, etc.). Lastly, participants were asked whether they used a computer, a tablet and/or a smartphone.

### Statistical analysis

Descriptive statistics were performed describing the sociodemographic characteristics of the included sample. Moreover, descriptive statistics on ICT and internet use were differentiated according to CF level (CFAI-COG).

Confirmatory factor analysis (CFA) was used to verify whether the IU frequency item, together with the nine IU activity items, was related to one latent concept (i.e. ‘internet use’). Then, the CFA model was included in a structural equation modelling (SEM) framework. A polychoric correlation matrix was used in combination with a diagonally weighted least squares (DWLS) estimator to obtain estimates of the constructed model. The CFA model was used to confirm the factor structure where the Chi-square (χ^2^), root mean square error of approximation (RMSEA), standardized root mean squared residual (SRMR), comparative fix index (CFI) and Tucker–Lewis Index (TLI) were used as fit indices to evaluate the model fit. The thresholds used to validate an acceptable model fit were RMSEA < 0.08, SRMR < 0.08, CFI > 0.95 and TLI > 0.95 (Hu and Bentler, 1999).

A linear regression analysis was integrated in the SEM procedure, with ‘internet use’ as dependent variable and all sociodemographic and CFAI-Plus domain scores as independent variables. To model more precise beta estimates, relationships between the different CFAI-Plus domains and the variable ‘age’ were explicitly modelled using the covariance matrix.

All statistical analyses were computed with RStudio 1.3.1093 running on R version 4.0.3 (R Core Team, 2020) using the lavaan (latent variance analysis) package. Statistical significance was accepted at *p* < 0.05.

## Results

### Descriptive results

Table 1 depicts sociodemographic data of the study sample. The sample consisted of 3019 community-dwelling older adults of a wide range of ages (60–103) and adequately represented the gender-balance of the population with 51.88% females. The majority has had at least lower secondary education and is currently living with a partner.

**Table 1:** Sociodemographic data of the participants (*n* = 3019)

Variables	n(%)^a^
Gender (*n* = 3009)	
Male	1448 (48.12%)
Female	1561 (51.88%)
Age (*n* = 3006)	
median ± IQR (min–max)	70.0 ± 13 (60–103)
Educational level (*n* = 2919)	
None or primary education	514 (17.61%)
Lower secondary education	838 (28.71%)
Higher secondary education	755 (25.87%)
Higher education	812 (27.82%)
Living situation (*n* = 3019)^b^	
Living alone^c^	543 (17.99%)
Not living alone	
Living with partner	2186 (72.41%)
Living with child(ren)	419 (13.88%)
Living with grandchild(ren)	47 (1.56%)
Living with others (parents or others)	196 (6.49%)
Net income (*n* = 2554)	
< €1250 per month	368 (14.41%)
€1250–2000 per month	901 (35.28%)
> €2000 per month	1285 (50.32%)

*IQR* interquartile range.

^a^Proportions are calculated based on the total number of cases that responded to this question. ^b^Multiple answers could be selected. ^c^People were considered living alone when not living together with partner, children, grandchildren, parents or others

Participants scored mainly no-low to mild on all CFAI-Plus subdomains (Table 2). The highest relative frequency was observed for CFAI-COG, with little less than half of the participants (46.09%) reporting mild-to-high CF symptoms, suggesting the presence of at least one subjective cognitive complaint.

**Table 2:** Comprehensive frailty assessment instrument (CFAI-Plus) data of the participants

CFAI-Plus domain	Total (*n* = 3019)
CFAI-COG (*n* = 2858)	
No-Low (0.00–3.13)^a^	1541 (53.92%)
Mild (4.69–9.38)^a^	855 (29.92%)
High (10.94–25.00)^a^	462 (16.17%)
CFAI-PHYS (*n* = 2698)	
No-Low (0.00–6.25)^b^	1945 (72.09%)
Mild (9.38–18.75)^b^	448 (16.60%)
High (21.88–25.00)^b^	305 (11.30%)
CFAI-PSY (*n* = 2758)	
No-Low (0.00–5.00)^b^	1870 (67.80%)
Mild (5.01–11.50)^b^	673 (24.40%)
High (11.51–25.00)^b^	215 (7.80%)
CFAI-SOC (*n* = 2653)	
No-Low (0.00–9.40)^b^	2099 (79.12%)
Mild (9.41–16.00)^b^	502 (18.92%)
High (16.01–25.00)^b^	52 (1.96%)
CFAI-ENV (*n* = 2818)	
No-Low (0.00–1.25)^b^	1671 (59.30%)
Mild (2.50–7.50)^b^	803 (28.50%)
High (8.75–25.00^b^	344 (12.21%)

CFAI-COG = CFAI cognitive frailty; CFAI-PHYS = CFAI physical frailty; CFAI-PSY = CFAI psychological frailty; CFAI-SOC = CFAI social frailty; CFAI-ENV = CFAI environmental frailty.

^a^Cut-off as proposed by De Roeck et al. (2018). ^b^Cut-off as proposed by De Witte et al. (2018)

The majority of the participants (77.57%) used the internet at least from time to time, with most of the participants using the internet at least daily (Table 3). The most frequent carried out activities were navigating the internet, sharing email and online banking. Internet was the least used for tele-communicating with Skype, online shopping and using e-government services. Computers and smartphones were more frequently used than tablets. When differentiating these results according to CF profile, more non-users were observed in the high CF group. The pattern of internet activities did not vary across CF profiles, but all internet activities were around 5–20 per cent less executed in the high CF group as compared to the no-low CF group. With regard to the used ICT devices, older adults with higher CF profiles used tablets less frequently compared to older adults with no or minor CF symptoms.

**Table 3:** ICT and internet use-related data of participants

	Complete sample	No-low CF^a^	Mild CF^a^	High CF^a^
ICT use, n(%)^b^				
Computer (*n* = 2375), Yes/Total number of ICT users	1333/1375 (96.95%)	798/816 (97.79%)	363/377 (96.29%)	139/145 (95.86%)
Smartphone (*n* = 1438), Yes/Total number of ICT users	1368/1438 (95.13%)	811/845 (95.98%)	368/396 (92.93%)	147/152 (96.71%)
Tablet (*n* = 1041), Yes/Total number of ICT users	924/1041 (88.76%)	582/641 (90.80%)	225/269 (83.64%)	87/97 (89.69%)
Internet use				
Frequency, n (%)	*n* = *3019*^*c*^	*n* = *1541*	*n* = *855*	*n* = *462*
Never	677 (22.42%)	186 (12.07%)	204 (23.86%)	206 (44.59%)
Less than weekly	139 (4.60%)	56 (3.63%)	45 (5.26%)	32 (6.93%)
Weekly	240 (7.95%)	115 (7.46%)	71 (8.30%)	39 (8.44%)
Daily	1104 (36.57%)	613 (39.78%)	321 (37.54%)	127 (27.49%)
Multiple times a day	859 (28.45%)	571 (37.05%)	214 (25.03%)	58 (12.55%)
Type of internet activity	*n* = *2342*^*d*^	*n* = *1355*	*n* = *651*	*n* = *256*
Navigating/searching the internet	2136 (91.29%)	1272 (93.87%)	588 (90.32%)	221 (86.33%)
E-mail	2028 (86.59%)	1219 (89.96%)	546 (83.87%)	208 (81.25%)
Online banking	1641 (70.07%)	1014 (74.83%)	443 (68.05%)	145 (56.64%)
Maintaining contact with (grand)children	1324 (56.53%)	781 (57.64%)	367 (56.37%)	137 (53.52%)
Social media (Facebook, twitter,…)	982 (41.93%)	596 (43.99%)	267 (41.01%)	92 (35.94%)
Online administration (registering subscriptions, buying tickets…)	930 (39.71%)	617 (45.54%)	235 (36.10%)	63 (24.61%)
E-government services	860 (36.72%)	573 (42.29%)	215 (33.03%)	54 (21.09%)
Online shopping	478 (20.41%)	544 (40.15%)	209 (32.10%)	64 (25.00%)
Skype	478 (20.41%)	294 (21.70%)	130 (19.97%)	43 (16.80%)

*CF* cognitive frailty.

^a^Cognitive frailty as measured with the CFAI-Plus. ^b^Because of missing data, relative proportion of participants responding with Yes to these questions were notated as fractions with the total number of answers as the denominator. ^c^Missing data on CF in *n* = 161. ^d^Missing data on CF in *n* = 80

### SEM

**Fig. 1:**
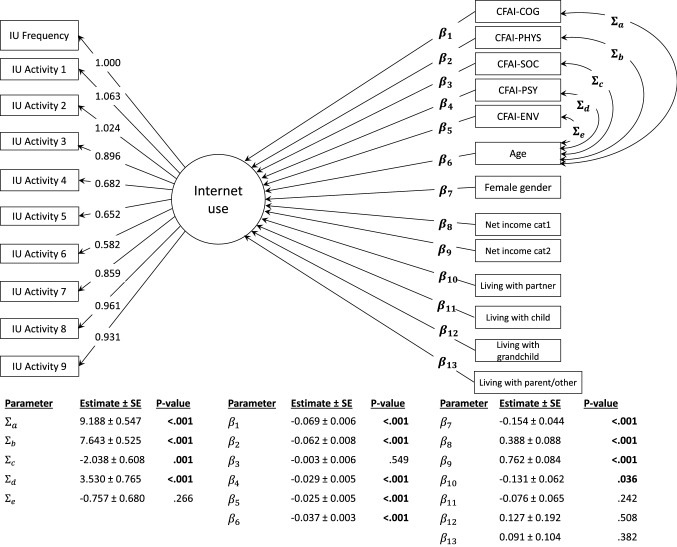
Fitted SEM model for ‘internet use’ (IU), including IU frequency and IU activity. *Note.* IU Activity 1 = navigating/searching the internet; IU Activity 2 = email; IU Activity 3 = e-government services; IU Activity 4 = maintaining contact with (grand) children; IU Activity 5 = social media; IU Activity 6 = Skype; IU Activity 7 = online shopping; IU Activity 8 = online banking; IU Activity 9 = online administration; Net income cat1 = €1250–2000; Net income cat2 =  > €2000

To determine whether the IU frequency item and the IU activity items were related to one latent concept (i.e. internet use), a CFA was performed. The estimate, through the diagonally weighted least squares (DWLS), showed adequate robust fit indices (χ^2^ = 1953.197, df = 204, *p* < 0.001; CFI = 0.967; TLI = 0.962; RMSEA = 0.064; SRMR = 0.061) to the model fitted in Fig. 1.

For the latent variable ‘internet use’, the highest predicted factor score corresponded with the combination of more frequent internet use (i.e. ‘daily’) and using the internet for all activities. The lowest predicted factor scores, on the other hand, represented the lowest frequency level (i.e. ‘never’) and the absence of internet activities. A total of 191 distinct activity patterns were identified from the IU activity data.

With regard to the modelled covariances, statistically significant relationships were observed between age and CFAI-COG (${\Sigma }_{a}$, *p* < 0.001), CFAI-PHYS (${\Sigma }_{b}$, *p* < 0.001), CFAI-SOC (${\Sigma }_{c}$, *p* = 0.001) and CFAI-PSY (${\Sigma }_{d}$, *p* < 0.001). For CFAI-ENV, no significant correlation with age was observed (${\Sigma }_{e}$, *p* = 0.266).

The linear regression model integrated in the SEM showed statistically significant lower levels of internet use for older age (${\beta }_{6}$, *p* < 0.001), female gender (${\beta }_{7}$; *p* < 0.001), lower net income (${\beta }_{8}$ and ${\beta }_{9}$, *p* < 0.001), living with a partner (${\beta }_{10}$, *p* = 0.036) and higher CFAI-Plus frailty scores in all domains, except for CFAI-SOC (${\beta }_{1}, {\beta }_{2}, {\beta }_{4}$ and ${\beta }_{5}$, *p* < 0.001; ${\beta }_{3}$, *p* = 0.549) (Fig. 1). An increase of one point on the CFAI-COG domain score resulted on average in a decrease of 0.069 (± 0.006) on the computed factor score for ‘internet use’ (${\beta }_{1}$, *p* < 0.001), when adjustments for all other covariables in the model were made. These covariables comprised: gender, age, net income, living with a partner, living with children, living with grandchildren, living with parents or others and all other CFAI-Plus domain scores.

## Discussion

This study aimed to determine the relationship between sociodemographic and multidimensional frailty factors, including CF, and internet use among older adults. Overall, internet use was high and frequent in our sample. This is in line with recent research findings (Stockwell et al. 2020; Vulpe and Crăciun, 2020) and findings of König et al. (2018) and indicates that Belgian older adults are also amongst the most frequent internet users in Europe. The most frequently executed activities were navigating the web, sharing email and online banking, whereas tele-communication, online shopping and consulting e-government services were the least common. This activity pattern was expected, based on previous research (Chang et al. 2015; Choi and DiNotto, 2013; Hernández-Encuentra et al. 2009; LaMonica et al. 2017). With regard to the use of e-government services among older adults, research is scarce and shows contradicting results (Bélanger and Carter, 2009; Flowers-Henderson, 2019). In the present study, older adults used more computers and smartphones than tablets. This was also in line with previous research (Chang et al. 2015; LaMonica et al. 2017). Although it is known that older adults are slower to go online or adopt new technologies (Anderson and Perrin 2017; Perrin and Atske 2021), according to the Diffusion of Innovation theory (Rogers 2003), it is likely that they will eventually catch up.

The most important finding was the inverse relationship between CF status and internet use. Moreover, the type of ICT device differed between the CF groups, with less tablet use in the higher cognitively frail older adults than in the other groups. A possible explanation for this finding is that people who are more cognitively impaired may find it more difficult to adopt to internet and new technologies, on top of cohort-related barriers. This is in accordance with previous studies that observed negative effects of lower cognitive ability on ICT and internet use (Choi and DiNotto 2013; Czaja et al. 2006; Elliot et al. 2013; Huxhold et al. 2020; LaMonica et al. 2017). However, often these studies included younger adults or defined cognitive ability in the absence of a formal diagnosis of cognitive decline or subjective memory complaints (Berner et al. 2013; Czaja et al. 2006; Elliot et al. 2013; Huxhold et al. 2020). Since the present study was able to differentiate in degree of CF, its results may be compared to those of Malinowsky et al. (2017) and LaMonica et al. (2017). Similar to these studies, our study findings show that CF is related to less internet use, less ICT use and less social media use. Moreover, internet and technology use decreased with increasing CF status. Given that higher CF older adults in the study of LaMonica et al. (2017) did not experience more difficulties using the internet, lower ICT and internet use among CF older adults could potentially be the result of unequal access, unsuccessful adoption or differences in attitude.

Since CF is associated with other frailty measures, the present study also included measures of physical, social, psychological and environmental frailty. The majority of these were also inversely related to internet use. First, more severe limitations in activities of daily living, reflected by the physical frailty factor, were associated with less internet use. These findings are comparable to those of Keränen et al. (2017) who also demonstrated a negative interaction between physical frailty and ICT use. This negative relationship was in the present study also observed for psychological frailty symptoms. These findings are in line with those of Choi and DiNotto (2013) in which depressive symptoms were associated with less internet use. Moreover, environmental frailty, comprising aspects of housing and neighbourhood, was also negatively associated with internet use. Likely, this frailty domain is interrelated to socioeconomic status and is therefore indirectly associated with internet use. For social frailty, no significant relationship with internet use could be observed. This was opposed to previous research that demonstrated facilitating effects of frequent social contacts and internet use among these important others (Choi & DiNotto 2013; Huxhold et al. 2020; König et al. 2018) as well as of social loneliness (Sum et al. 2008). Moreover, positive associations between internet use and informal and formal participation in older adults have already been demonstrated (Pan et al. 2019). However, since dispersion of the CFAI-SOC variable was low with the majority of participants scoring no-low to mild, the absence of a statistically significant result was to be expected.

Although internet access and use have increased among older adults, narrowing the so-called grey digital divide (Huxhold et al. 2020; Morris 2007), our analysis showed that older age is still negatively related to internet use. This was expected, as this is in line with the previous research (Arcury et al. 2018; Chang et al. 2015; Choi and DiNotto 2013; König et al. 2018). Possibly, the lack of adequate previous workplace experience with technology as well as generational differences in consumer behaviour results in the differentiation of age-cohorts with regard to internet use (Choi and DiNotto 2013; Gilleard and Higgs 2008; König et al. 2018). This suggests that the double greying effect still has its implications for internet use among the older adult population. Furthermore, lower net income was associated with less internet use. As previous research has already frequently demonstrated the comprehensible relationship between socioeconomic status and internet use among older adults, this result was also expected (Arcury et al. 2018; Choi and DiNotto 2013; Freese et al. 2006; König et al. 2018). The female gender was also negatively related to internet use, which is in line with previous research findings (Choi and DiNotto 2013; König et al. 2018) and could also potentially be explained by less previous workplace experience with technology. Therefore, this gender gap is believed to be a transient phenomenon (Huxhold et al. 2020; König et al. 2018). Lastly, living with a partner was negatively associated with internet use. This was unexpected, since previous research has proven that being married or living with a partner is positively related to internet use in older adults (Arcury et al. 2018; Choi and DiNotto 2013). However, it is conceivable that older adults who live with a partner experience less need for digital activities or engagement because they feel sufficiently connected to or supported by them (Peek et al. 2016).

The present study used a large sample of community-dwelling older adults and was able to examine the relationship between internet use and CF in this population from a multidimensional perspective. Therefore, we integrated sociodemographic and CF measures in a comprehensive explanatory model. Although the present study’s main focus was on CF, multiple relevant other frailty measures were at our disposal and were integrated in the analysis. Therefore, the present study distinguishes itself from other studies in the field and can inspire future research in this domain. Moreover, in contrast to the binary measurement (yes/no) of internet use among older adults in many previous studies (Choi and DiNotto 2013; Huxhold et al. 2020; Kamin and Lang 2020; König et al. 2018), the present study used a more fine-grained approach by measuring internet use frequency with a 5-point Likert scale. However, the present study has some potential limitations to acknowledge. We estimated CF exclusively by means of subjective cognitive complaints and did not make use of objective measures of global cognitive status. Although research has already delivered considerable evidence for the strong relationship between subjective cognitive measures and the occurrence of cognitive decline and neurodegenerative diseases (Jessen et al. 2014; Koppara et al. 2015; Perrotin et al. 2017), our research findings can, however, only confidently be applied to the population of older adults with SCI. Furthermore, as we did not use objective cognitive measures, nor excluded participants based on formal diagnoses, it is not completely impossible that the present study has included participants with more major cognitive decline, as is the case for MCI or dementia. However, this is very unlikely, as filling in the survey requires having relatively good cognitive abilities. Moreover, up until now, the BAS survey consists of a limited number of items referring to internet use. Hence, it does not include aspects related to accessibility or acceptance of technology, which are also important factors in internet use amongst older adults (Peek et al. 2016). However, since the BAS survey consists of several other relevant items, it was of specific value to the multidimensional approach of the present study. Furthermore, since the present study applied a cross-sectional design, changes in internet and ICT use, and thus in internet use, could not be detected. Moreover, detection of potential changes in use behaviour is of utter importance, since technology use in older adults can be subject to disruption (Peek et al. 2019). As a consequence, internet use among older adults should be considered as a dynamic process that is regulated by a complex interplay of influencing factors. Lastly, due to the fast pace in which internet and technology adoption among all age groups is constantly increasing and the large time-frame in which the study data were gathered, the present study’s findings will potentially need an update over time.

Future research in this field should consider using other methodological approaches such as a more condense time-frame or prospective longitudinal designs, to adequately reflect the changing trends in internet use of older adults over the years. Since internet use is increasingly shifting towards mobile technology, future research in this domain should also focus more on these new media. In addition, research that focuses on other aspects than technology use behaviour, such as processes of acceptance and adoption, has a clear added value. This was already demonstrated by Peek et al. (2016, 2019). However, these aspects of internet use remain relatively unexamined in the cognitively frail older adult population specifically. The use of qualitative or mixed-method designs could be beneficial to get a deeper understanding of these processes in this population. Lastly, since digitisation of society is moving faster than ever due to the worldwide COVID pandemic, it is important to extend the research on internet use of services that are currently being increasingly developed, such as e-government services.

To conclude, the results of this study have shown that most older adults seem to use the internet, although not all internet activities are equally performed by them. Sociodemographic as well as cognitive, biological, psychological and environmental frailty factors are related to internet use and require specific attention when promoting internet use in older adult populations. Future research in this field should focus on the use of digital services, such as e-government services, that are understudied but that are likely of increasing social importance. Moreover, it is directed towards the use of methodological approaches that emphasize the complex and dynamical processes underlying internet use in the cognitively frail older adult population.

## Supplementary Information

Below is the link to the electronic supplementary material.

Supplementary file1 (DOCX 17 kb)

## Data Availability

Not applicable.
