# Supplementary material for: Internet use and cognitive frailty in older adults: a large-scale multidimensional approach
Source: Eur J Ageing. 2022 Mar 2;19(4):1135–44. doi: 10.1007/s10433-022-00686-2 (PMC9729622; doi:10.1007/s10433-022-00686-2)
Supplement: Supplementary file 1 — Supplementary file1 (DOCX 17 kb) [file 10433_2022_686_MOESM1_ESM.docx]

| **Supplementary file 1** CFAI-Plus domains with their items | |
| --- | --- |
| CFAI-Plus domain | Scale |
| CFAI-COG |  |
| To what extent do you agree with the following statements:   1. I have trouble with remembering things that have happened recently. 2. I experience difficulties with learning new things in general. 3. I experience difficulties with handling financial matter, e.g., the pension, dealing with the bank. 4. I have trouble with following a story in a book or in a TV. | 1=totally disagree  2=disagree  3=neither agree nor disagree 4=agree  5=totally agree |
| CFAI-PHYS |  |
| Have the following activities been hampered by your state of health, if so, for how long? (please tick all appropriate items)   1. Less demanding activities like carrying shopping bags 2. Walking up a hill or some stairs 3. Bending or lifting 4. Going for a walk | 1= not at all 2=3 months or less 3=more than 3 months |
| CFAI-SOC |  |
| *Social loneliness*  To which extent do you agree with the following statements:   1. There are enough people whom I can rely on when I am in trouble. 2. I know many people whom I can totally trust. 3. There are enough people with whom I feel a bond.   *Social support network*  Suppose you are unable to carry out the activities you usually do in the housekeeping for a certain while, whom would you be able to appeal to? (please tick all appropriate items, various answers may be given)   1. Partner 2. Daughter 3. Son 4. Daughter in law 5. Son in law 6. Grandchild or great grandchild 7. Sister or brother (sister-in-law/brother-in-law) 8. Other family member 9. Neighbour 10. Friend/acquaintance 11. No one | 1=totally disagree  2=disagree  3=neither agree nor disagree  4=agree  5=totally agree |
| CFAI-PSY  *Mood*  Considering the last few weeks, to which extent do you agree with the following (please tick):   1. I feel unhappy and depressed. 2. I feel like I’m losing my self-confidence. 3. I feel like I cannot cope with the problems. 4. I feel like I’m under constant pressure. 5. I feel like I’m not worth anything anymore.   *Emotional loneliness*  To which extent do you agree with the following statements (please tick all appropriate items):   1. I experience a general sense of emptiness. 2. I miss having people around me. 3. I often feel rejected | 1=not at all  2=not more than usual  3=more than usual  4=considerably more than usual  1=totally disagree  2=disagree  3=neither agree nor disagree  4=agree  5=totally agree |
| CFAI-ENV |  |
| Which statements are applicable to your house? (please tick all appropriate items)   1. The house is in a bad condition/poorly kept. 2. The house is not very comfortable. 3. It is difficult to heat the house. 4. There is insufficient comfort in the house. 5. I do not like the neighbourhood. | 1=not applicable at all  2=rather not applicable  3=neither applicable nor inapplicable  4=rather applicable  5=completely applicable |
| *Note.* CFAI-COG=Cognitive CFAI-Plus domain; CFAI-PHYS=Physical CFAI-Plus domain; CFAI-SOC=Social CFAI-Plus domain; CFAI-PSY=Psychological CFAI-Plus domain; CFAI-ENV=Environmental CFAI-Plus domain. | |
